# Enhanced load frequency control using a novel fractional-order integral-integral-derivative controller optimized by cuckoo catfish optimizer

**DOI:** 10.1038/s41598-026-52025-5

**Published:** 2026-06-11

**Authors:** Mohamed Barakat, Ahmed Donkol, Mohammed Sekhi, A. M. Mabrouk

**Affiliations:** 1 Electronics and Communication Engineering Department, Giza Engineering Institute, Giza, Egypt; 2 Computer Systems Engineering, Faculty of Engineering, MSA University, 6-October, Giza, Egypt; 3 Department of Electrical Engineering, Faculty of Engineering, Qena University, Qena, Egypt; 4 Department of Computer Science, Shatt Al-Arab University College, Basra, Iraq; 5 Faculty of Engineering, King Salman International University (KSIU), South Sinai, El Tur, 46612 Egypt

**Keywords:** Fractional-order integral integral derivative (FOIID) controller, Integral time absolute error (ITAE), Harris Hawks optimizer, Generation rate constraint nonlinearities, Hydrothermal interconnected power system, Energy science and technology, Engineering, Mathematics and computing

## Abstract

Maintaining frequency stability in interconnected power systems (IPSs) is a critical challenge, particularly under sudden load changes and nonlinear constraints. Conventional PID and fractional-order controllers (FOPID, TID, FOID, and cascaded FO-PID structures) either lack adaptability or introduce excessive complexity. To overcome these limitations, this study introduces a novel fractional-order integral–integral–derivative (FOIID) controller that replaces the proportional term with a second-order fractional integrator. This dual-integral design yields 27 possible configurations, enhances the low-frequency gain, and eliminates the steady-state ramp error, thereby improving the transient and steady-state performance. The recently developed cuckoo catfish optimizer (CCO) is employed for parameter tuning. Unlike conventional metaheuristics (PSO, GA, and GWO), CCO integrates cooperative space compression, chaotic predation, and adaptive regeneration strategies, which avoid premature convergence and achieve a robust global search. The proposed CCO–FOIID framework was validated on a two-area non-reheat benchmark system and further tested on a three-area thermal–thermal–hydro system with generation rate constraints under 1% and 2% step load disturbances. A comparative analysis against five state-of-the-art controllers (ISFS–PID, DSA–FOPID, WHO–PI(1 + FOPID), and CGO–FOPID–FOPI) demonstrates that CCO–FOIID consistently achieves faster settling times, reduced overshoot, and the lowest ITAE value (74.26), outperforming the best competitor (CGO–FOPID–FOPI, 82.67). These results confirm that the combination of FOIID’s universal structure and CCO’s robust optimization provides a simple yet powerful solution for modern LFC applications in both simple and complex IPS networks.

## Introduction

In modern IPSs, the continuous alignment of power generation with consumer demand is essential. Frequency deviations caused by mismatches adversely affect the device performance and necessitate robust control mechanisms. The LFC evaluates system stability under varying SLCs, striving to maintain the frequency and tie-line power at nominal values. However, the variable nature of power demand, compounded by nonlinearities and uncertainties, exacerbates instability issues^1,2^.

Numerous control methodologies have been proposed to address LFC challenges, including optimal, adaptive, and artificial intelligence (AI)-based approaches^3^. Conventional PI and PID controllers are widely used because of their simplicity, reliability, and cost-effective implementation^4–6^. However, their performance deteriorates when operating points deviate significantly from their design conditions. However, their performance deteriorates when the operating points deviate significantly from the design conditions. To overcome these limitations, advanced control techniques have been explored, such as sliding mode control^7^, fuzzy logic control^8,9^, linear matrix inequality methods^10^, internal model control (IMC)^11^, optimal control^12^, model predictive control^13^, and variable structure control^14^. Despite their potential, these advanced methods are often complex and are therefore less frequently adopted in industrial applications^15^.

Recently, fractional-order controllers (FOCs), particularly FOPID controllers, have attracted significant research attention due to their flexibility, performance, robustness, and suitability for complex systems such as IPSs, including renewable energy sources^16–18^. In^19^, an improved method was proposed for regulating the frequency of a two-area power system (PS) using a cascade configuration that combines a tilt integral–derivative (TID)-FOPID with a filter controller. The ability to incorporate memory effects and effectively handle nonlinearities makes these networks powerful tools in modern control engineering. Consequently, several FOPID-based structures have been developed, including cascaded configurations such as FOPID-FOPI, FOPI-FOPD, FOPI-FOPTID, PI^λ^ (1 + PDF), and PI (1 + FOPID), as suggested in^1,20–23^. However, despite their enhanced performance, cascaded controllers remain complex and highly sensitive to parameter tuning, which limits their practical implementation and necessitates a meticulous design to fully realize their potential.

To refine the controller parameters in modern PSs, numerous soft computing techniques have been employed, including the artificial bee colony algorithm^24^, cuckoo search optimization^25^, Harris Hawks optimization (HHO)^26^, particle swarm optimization (PSO)^27^, and grey wolf optimization (GWO)^28^, among others. In addition, hybrid approaches have been developed to achieve smooth deviations, such as harmony search combined with cuckoo search^29^ and the integration of artificial bee colony with mine blast algorithms^25^, have been developed to further improve system stability by minimizing frequency and tie-line power deviations, particularly in the context of LFC. Although these metaheuristic algorithms significantly enhance the transient response and overall PS stability, the no free lunch theorem underscores the necessity of exploring novel optimizers^15^. Therefore, the application of recent and advanced optimization algorithms is essential to further improve the LFC performance and adaptability in evolving PS environments.

In summary, advancements in AGC and LFC performance are increasingly driven by soft computing–based controller designs. However, prior studies reveal persistent limitations: classical PI/PID controllers lack robustness under nonlinearities, advanced controllers such as MPC and sliding mode are computationally complex and rarely adopted in practice, cascaded fractional-order structures are highly sensitive to parameter tuning, and conventional metaheuristics often suffer premature convergence. These gaps highlight the need for approaches that combine simplicity and robustness. By providing adaptive and cost-effective solutions, soft computing–based designs ensure stable and efficient grid operations. Superior grid stability depends on three key elements: an advanced controller structure, a robust optimization algorithm, and a well‑formulated cost function. Motivated by these limitations, this study introduces the FOIID controller and CCO optimizer as a compact yet powerful framework for modern IPSs.

In this study, a novel controller structure, the fractional-order integral–integral–derivative (FOIID) controller, is proposed and compared with various single and cascaded controller structures under identical operating conditions. Unlike existing fractional-order controllers, the FOIID replaces the proportional term with a second fractional integrator. This dual-integral design yields 27 distinct configurations, enhances the low-frequency gain, and uniquely eliminates the ramp error. FOIID achieves the performance of cascaded fractional controllers in a single compact structure, requiring only six tunable parameters. This balances robustness and simplicity, making it more practical for real-world LFC applications. Furthermore, a recent and robust metaheuristic optimizer, the cuckoo catfish optimizer (CCO)^31^, was employed. CCO mitigates premature convergence and enhances population diversity by mimicking catfish predation strategies, including space compression, chaotic invasion, and survival-based regeneration, thereby improving the global search capability. Consequently, a CCO-optimized FOIID controller is proposed to address the LFC challenges in modern power systems.

This study advances AGC/LFC research in IPSs through the following key contributions.

i. Novel controller structure: An FOIID controller is proposed as a universal structure with 27 possible configurations, enabling flexible adaptation to diverse IPSs conditions.
ii. Advanced optimization strategy: The recent and robust CCO is employed to tune the controller parameters, leveraging its strong global search capability and enhanced convergence behavior to avoid premature stagnation.
iii. Fair benchmarking: The corresponding integral of time-weighted absolute error (ITAE) values was calculated to enable a rigorous and fair comparison with state-of-the-art methods.
iv. Validation and comparative analysis: The CCO-optimized FOIID controller is applied to a standard two-area non-reheat PS to evaluate the individual and synergistic effectiveness of the approaches.
v. Robustness demonstration: A challenging three-area thermal–hydro system with generation rate constraint (GRC) nonlinearity is developed to demonstrate the robustness of the proposed scheme under realistic physical constraints.

## Materials and methods

### Overview of LFC

**Fig. 1:**
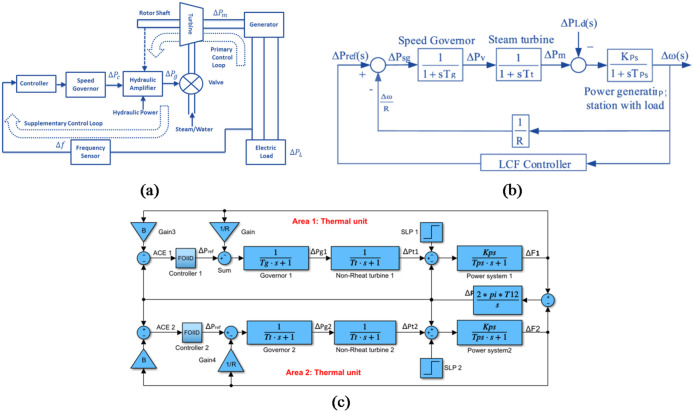
(a) Basic LFC scheme; (b) transfer function model of single area; (c) transfer function model of two area IPS.

The AGC approach employs two control loops: a primary loop that provides a fast response and a secondary loop that ensures long-term stability and precise frequency regulation^30^. As shown in Fig. 1(a), components such as the speed valve controller, turbine, generator, and governor were integrated into a single-area model. Frequency deviation responds to load changes and is compared with a reference speed setting to adjust the steam valve, aligning generator power with consumer demand and restoring frequency to its nominal value. However, owing to physical constraints, primary frequency control responses are delayed, necessitating a secondary controller to enhance system stability. The model shown in Fig. 1(a) is mathematically represented in Fig. 1(b). The core components, namely the governor, turbine, and generator load system, are characterized by the following transfer functions (TFs)^21^:

- Governor model: The governor time constant is denoted by, and Its TF is expressed as.

1 $${G_G}\left( s \right)=\frac{1}{{1+{T_{sg}}S}}$$

- Turbine model: The turbine converts the potential energy of pressurized steam or water into kinetic energy, which is subsequently transformed into electrical energy by a generator. Its TF is given by:

2 $$~{G_T}\left( s \right)=\frac{1}{{1+{T_{st}}s}}$$

- Generator-load model: The combined generator and load dynamics are represented by the following TF:

3 $${G_L}\left( s \right)=\frac{{{K_{gs}}}}{{1+{T_{gs}}s}}$$

The complete system model integrates the primary and secondary control loops with the LFC controller to ensure stability and precise frequency regulation.

**Fig. 2:**
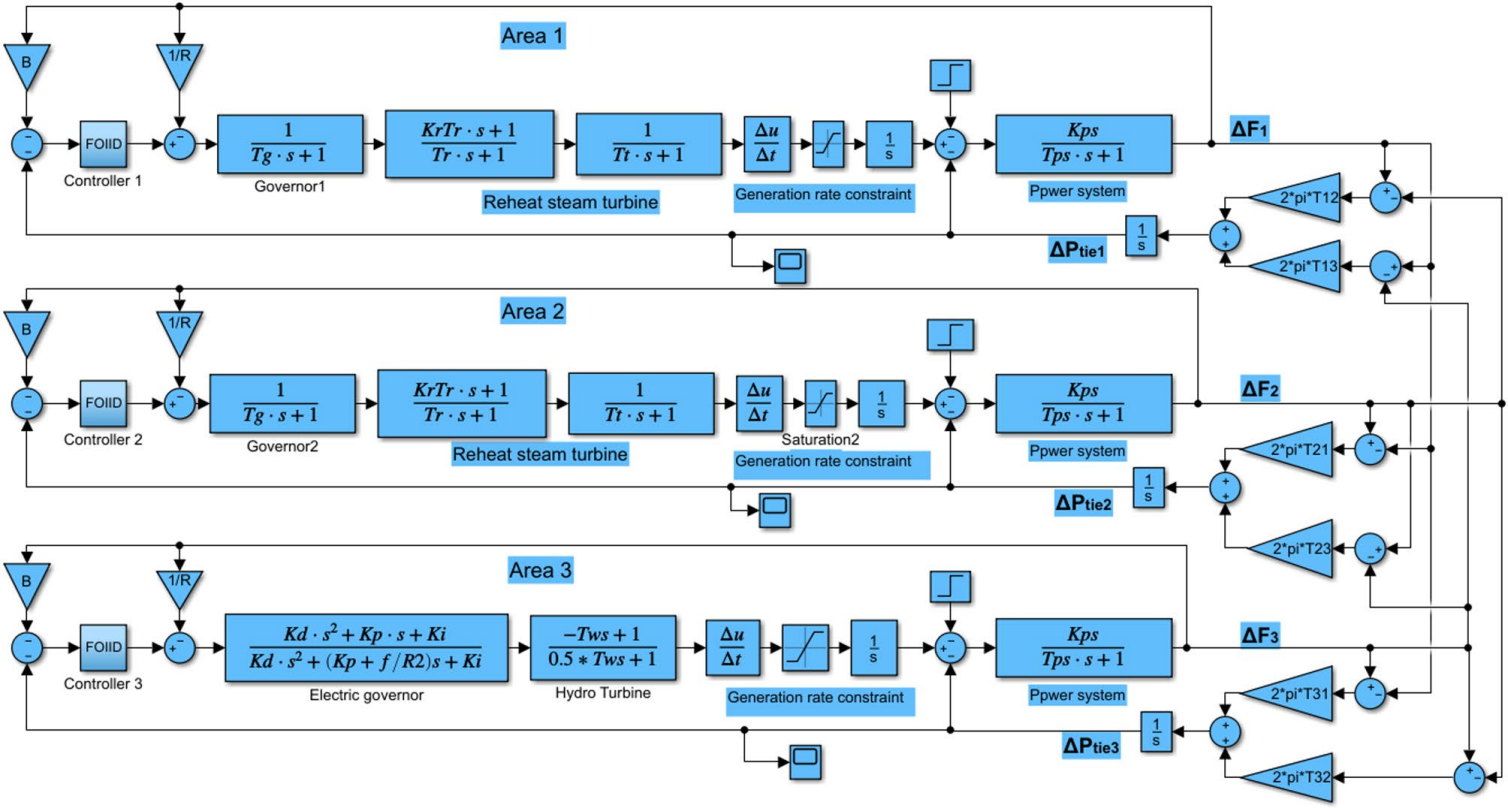
TF model of the thermal hydro system.

**Fig. 3:**
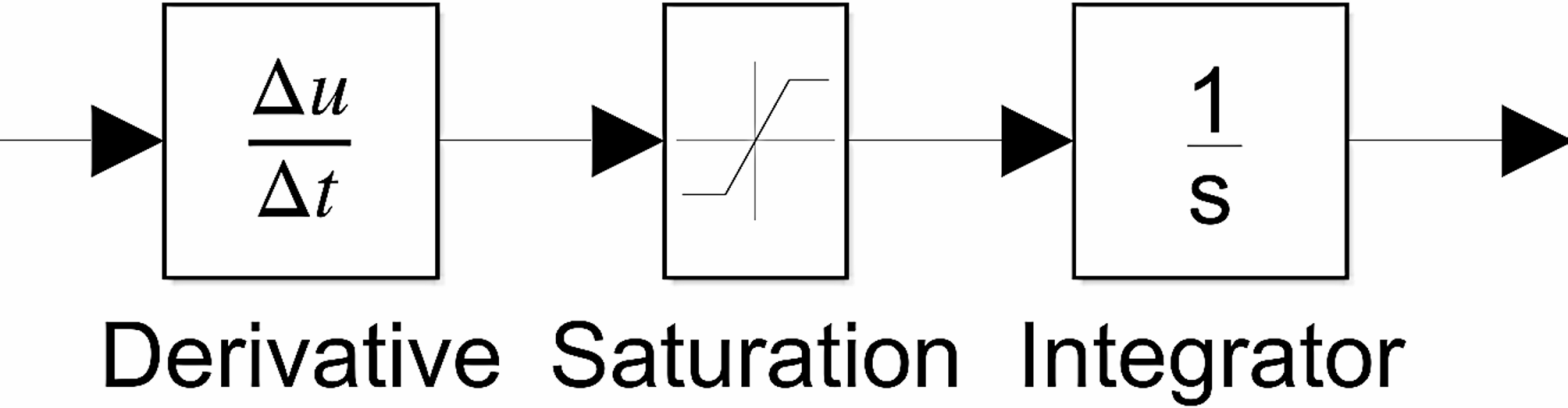
Modeling of GRC nonlinearity.

### Two area non reheat PS

Building on the single‑area model, the complete representation of a two‑area IPS with non‑reheat units in each area is depicted in Fig. 1(c). This model is a common test system in literature. The proposed FOIID controller is employed as a secondary controller, incorporating both the frequency bias factor and tie‑line interconnection. The frequency bias factor (*B*), defined as the sum of the frequency‑sensitive load change (*D*) and the inverse of speed regulation (*R*), is expressed as^31^:

4 $$~~B=\frac{1}{R}+D$$

The primary objective is to minimize the area control error (ACE), which captures the combined effects of frequency deviations and tie‑line power discrepancies. Minimizing the ACE ensures stable and equitable power sharing among interconnected regions, thereby promoting overall system stability^23,24^. The FOIID controller in each area uses ACE as its input. The ACEs for the two areas are defined as^24^:

$$~ ~AC{E_1}=\Delta {P_{{\mathrm{tie}}}}+{B_1}\Delta {f_1}$$

5 $$AC{E_2}={{\boldsymbol{\Delta}}}{P_{{\mathrm{tie}}}}+{B_2}{{\boldsymbol{\Delta}}}{f_2}$$

where $\Delta {P_{{\mathrm{tie}}}}$ is the tie-line power change, and $\Delta {f_{\mathrm{1}}}~$and $\Delta {f_{\mathrm{2}}}$ are the frequency changes in areas 1 and 2, respectively. When the system experiences a load change, the ACEs act as regulating signals to drive both ${{\boldsymbol{\Delta}}}{{\mathrm{P}}_{{\mathrm{tie}}}}$ and ${{\boldsymbol{\Delta}}}{{\mathrm{f}}_{\mathrm{I}}}$ toward zero. The detailed two‑area system parameters are provided in Appendix A.

### Three-area IPS

A three-area IPS, consisting of two thermal areas and one hydro area, was established as a challenging benchmark to evaluate the proposed approach for managing multi-area heterogeneous PSs, as shown in Fig. 2. Owing to physical and dynamic constraints, the generation cannot change instantaneously. To capture this limitation, GRC nonlinearities were applied to all areas, as illustrated in Fig. 3. For thermal plants, the GRC is typically 3% per minute, whereas for the hydro unit, it is 360% per minute for decreasing generation and 270% per minute for increasing generation^20^. Three FOIID controllers are required to control this complex system, each with multiple parameters that must be tuned simultaneously. Given the dimensionality of the problem, an advanced and recent optimization algorithm, such as CCO, is employed to efficiently adjust the controller parameters, ensuring effective frequency regulation and stable operation across the IPSs. The detailed three-area system parameters are presented in Appendix B.

### Proposed FOIID controller structure

#### Overview of PID, TID, and FOPID controllers

**Fig. 4:**
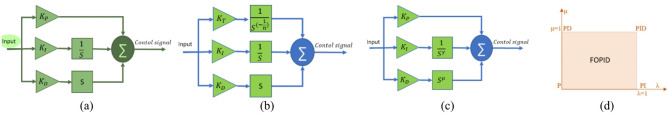
The controller structures: (a) PID, (b) TID, (c) FOPID, and (d) Plane of FOPID.

Conventional controllers such as PID, illustrated in Fig. 4(a), are widely adopted due to their simplicity, effectiveness, and versatility. However, despite their popularity, they exhibit inherent limitations when applied to the complex dynamics of modern power systems. Specifically, PID controllers struggle to precisely shape dynamic responses under significant disturbances and nonlinearities, including governor deadbands, generation rate constraints, and renewable energy variability^34^. The PID controller operates through three components: the proportional term generates a control action proportional to the current error, providing immediate response but potentially leaving steady‑state errors; the integral term eliminates steady‑state errors by accumulating past errors but may increase oscillations if improperly tuned; the derivative term predicts future error trends to reduce overshoot and stabilize the system but is sensitive to measurement noise. The TF of the PID controller is expressed as^4^:

6 $${G_{PID}}\left( s \right)=~{K_P}+\frac{{{K_I}}}{S}+{K_D}S$$

These limitations have driven the search for more advanced control strategies capable of addressing the dynamic and unpredictable nature of modern PSs. Control system engineering has witnessed a noticeable increase in the adoption of FOCs owing to their reduced response time, robust stability under varying conditions, and excellent resilience to external disturbances^35^. In a TID controller, the proportional term of the PID is replaced by the tilted factor, which improves set-point tracking and enhances disturbance rejection^36^, as shown in Fig. 4(b). The TF of TID controller is expressed as^36^:

7 $${G_{TID}}\left( s \right)=~\frac{{{K_T}}}{{{s^{\frac{1}{n}}}}}~+\frac{{{K_I}}}{S}+{K_D}S~$$

The parameter *n* in the TID controller typically lies within the range^2,5,37^. Several researchers have implemented TID controllers in cascade structures to improve LFC performance, including TD–TI, fuzzy TIDF, and ITDF controllers^38,39^.

Building on the concept of FOCs, the FOPID controller extends the conventional PID by introducing fractional orders for the integral and derivative terms. It is often referred to as the ${\mathrm{P}}{{\mathrm{I}}^{{\boldsymbol{\uplambda}}}}{{\mathrm{D}}^{{\boldsymbol{\upmu}}}}{\mathrm{~controller}}$, where $\lambda$ and $\mu$ are non-integer orders of the integral and derivative components, typically within the range [0, 1], as shown in Fig. 4(c). According to^20^, varying the $\lambda$ and $\mu$ yields nine distinct controllers (P, PI, PD, PID, P${{\mathrm{I}}^{{\boldsymbol{\uplambda}}}},~{\mathrm{P}}{{\mathrm{D}}^{{\boldsymbol{\upmu}}}},$ PI${{\mathrm{D}}^{{\boldsymbol{\upmu}}}},~{\mathrm{P}}{{\mathrm{I}}^{{\boldsymbol{\uplambda}}}}$D, and ${\mathrm{P}}{{\mathrm{I}}^{{\boldsymbol{\uplambda}}}}{{\mathrm{D}}^{{\boldsymbol{\upmu}}}}$), as illustrated in Fig. 4(d). The inclusion of tunable parameters allows flexible combinations of proportional, integral, and derivative actions. This adaptability enhances the robustness of the system while simultaneously improving its performance^22^. The TF of the FOPID controller is described as^1^:

8 $${G_{FOPID}}\left( s \right)={K_P}+\frac{{{K_I}}}{{{S^\lambda }}}+~{K_D}~{S^\mu }~$$

**Fig. 5:**
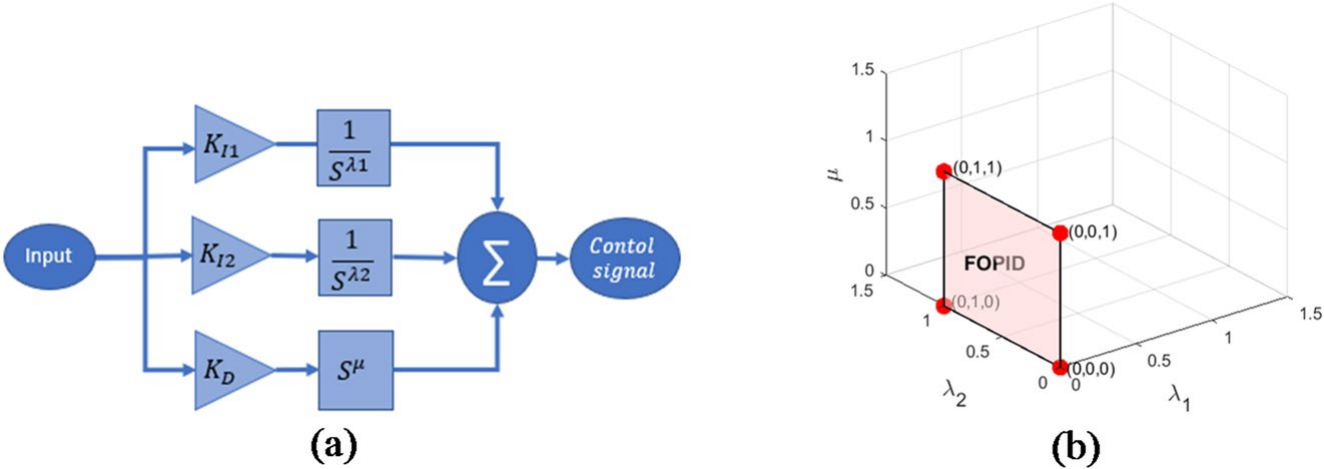
(a) Proposed FOIID controller structure and (b) The 3D visualization of the proposed FOIID controller.

#### Proposed FOIID controller

This study introduces a novel fractional‑order integral–integral–derivative (FOIID) controller, as illustrated in Fig. 5(a). The FOIID design synthesizes two established strategies.

(i) The non-integer integration and differentiation orders ($\lambda$ and $\mu$) of the FOPID controller,
(ii) The tilt factor from the TID controller, which acts as a fractional integrator within the range [0.2, 0.5] when n is substituted with values of 5 and 2, as reported in^37^.

In this study, the tilt factor was replaced by a fractional integrator with a dynamic range of [0, 1.5]. The TF of the proposed FOIID controller is expressed as^37^:

9 $${G_{FOIID}}\left( s \right)=\frac{{{K_{I1}}}}{{{S^{{\lambda _1}}}}}+\frac{{{K_{I2}}}}{{{S^{{\lambda _2}}}}}+{K_D}~{S^\mu }~$$

where ${\lambda _1}$, ${\lambda _2},$ and $\mu$ are the non-integer orders of the dual integral and derivative components, respectively, and ${K_{I1}}$, ${K_{I2}},~{\mathrm{and}}~{K_D}$ denote their corresponding gains. A 3D visualization of the tuning plane is shown in Fig. 5(b).

The two parallel fractional integrators generate the control input as a weighted sum of two fractional order error accumulations. This structure introduces additional degrees of freedom in the low-frequency dynamics, enabling the controller to simultaneously account for the fast and slow components of the frequency error trajectory. Consequently, the FOIID controller provides more effective attenuation of sustained frequency deviations than the single integral configuration. Furthermore, by eliminating the proportional term, the controller avoids the instantaneous control surge typically associated with step-like disturbances, thereby reducing the aggressive actuator effort, limiting the overshoot, and improving the damping of transient oscillations during frequency restoration.

By varying the non-integer orders, the FOIID structure generates 18 distinct controller configurations, as demonstrated in Table 1, excluding duplicates marked with (–). For instance, setting ${\lambda _1}=0~$recovers the FOPID controller, while zeroing all parameters yields simpler controller, e.g., P controller. The inclusion of configurations in which fractional integrators operate within [0.2, 0.5] expands the total to 27 achievable structures, establishing FOIID as a universal controller with three key advantages:

- Compact design is characterized by six tunable parameters.
- Enhanced adaptability through dual fractional-integral compensation for nonlinear dynamics.
- Superior performance with reduced settling times and peak deviations compared to conventional controllers.

The broad configurability demonstrated in Table 1; Fig. 5(b) positions the FOIID as a versatile solution for diverse applications.

Finally, in real-time applications and simulations, the fraction-order TFs must be approximated to integer-order equivalents. The Oustaloup approximation method is employed within a frequency band [${\omega _L},~{\omega _H}$], where ${\omega _L}$ is the lower frequency, and ${\omega _H}$ is the upper frequency, and N is the order of approximation. The formulation is given by^1^ as follows:

10 $${G_f}\left( s \right)={S^\alpha }=K\mathop \prod \limits_{{K= - N}}^{N} \frac{{s+\omega _{k}^{z}}}{{s+\omega _{k}^{p}}}~$$

Here, K is the filter gain, and $\omega _{k}^{p}$ and $\omega _{k}^{z}$ denote the pole and zero frequencies, respectively. The key implementation challenge is balancing the approximation accuracy (governed by N) with the computational complexity. Lower *N* values simplify processing but introduce ripples in the phase/magnitude responses. This study adopts $N=3$ with a frequency band of [${10^{ - 2}},~{10^2}$] ${\mathrm{rad}}/{\mathrm{s}}$^1^.

For instance, Barakat in^1^ proposed a simplified implementation scheme for fractional-order controllers using first- and second-order integer approximations, significantly reducing the computational overhead while preserving the control performance. The FOIID controller, with its six tunable parameters, can be deployed using similar approximation techniques.

**Table 1:** Controllers obtained by varying ${\lambda _1},~{\lambda _2}$ and µ at the FOIID controller.

	Fraction differentiator (µ)	Fraction integrator 2 (λ_2_)	Fraction integrator 1 λ_1_	Controller obtained	Literatured
1	0	0	0	*P*	^40^
2	0	0	1	PI	^41^
-	0	1	0	PI	-
3	0	1	1	P2I	^42^
4	0	0	${\lambda _2}$	P${{\mathrm{I}}^{{\boldsymbol{\uplambda}}}}$	f
5	0	1	${\lambda _2}$	P${\mathrm{I}}{{\mathrm{I}}^{{\boldsymbol{\uplambda}}}}$	NA
-	0	${\lambda _1}$	0	P${{\mathrm{I}}^{{\boldsymbol{\uplambda}}}}$	-
-	0	${\lambda _1}$	1	P${\mathrm{I}}{{\mathrm{I}}^{{\boldsymbol{\uplambda}}}}$	-
6	0	${\lambda _1}$	${\lambda _2}$	${\mathrm{P}}{{\mathrm{I}}^{{{{\boldsymbol{\uplambda}}}_1}}}{{\mathrm{I}}^{{{{\boldsymbol{\uplambda}}}_2}}}$	^43^
7	1	0	0	PD	^44^
8	1	0	1	PID	^45^
-	1	1	0	PID	-
9	1	1	1	2ID	^46^
10	1	0	${\lambda _2}$.	P${{\mathrm{I}}^{{\boldsymbol{\uplambda}}}}{\mathrm{D}}$	^47^
11	1	1	${\lambda _2}$	${\mathrm{I}}{{\mathrm{I}}^{{\boldsymbol{\uplambda}}}}{\mathrm{D}}$	NA
-	1	${\lambda _1}$	0	P${{\mathrm{I}}^{{\boldsymbol{\uplambda}}}}{\mathrm{D}}$	^48^
-	1	1	1	${\mathrm{I}}{{\mathrm{I}}^{{\boldsymbol{\uplambda}}}}{\mathrm{D}}$	-
12	1	${\lambda _1}$	${\lambda _2}$	${{\mathrm{I}}^{{{{\boldsymbol{\uplambda}}}_1}}}{{\mathrm{I}}^{{{{\boldsymbol{\uplambda}}}_2}}}{\mathrm{D}}$	NA
13	$\mu$	0	0	${\mathrm{P}}{{\mathrm{D}}^{{\mu }}}$	^20^
14	$\mu$	0	1	${\mathrm{PI}}{{\mathrm{D}}^{{\mu }}}$	^49^
-	$\mu$	1	0	${\mathrm{PI}}{{\mathrm{D}}^{{\mu }}}$	-
15	$\mu$.	1	1	$2{\mathrm{I}}{{\mathrm{D}}^{{\mu }}}$	NA
16	$\mu$	0	${\lambda _2}$	${\mathrm{P}}{{\mathrm{I}}^{{\boldsymbol{\uplambda}}}}{{\mathrm{D}}^{{\mu }}}$	^22^
17	$\mu$	1	${\lambda _2}$	${\mathrm{I}}{{\mathrm{I}}^{{\boldsymbol{\uplambda}}}}{{\mathrm{D}}^{{\mu }}}$	NA
-	$\mu$	${\lambda _1}$	0	${\mathrm{P}}{{\mathrm{I}}^{{\boldsymbol{\uplambda}}}}{{\mathrm{D}}^{{\mu }}}$	-
-	$\mu$	${\lambda _1}$	1	${\mathrm{I}}{{\mathrm{I}}^{{\boldsymbol{\uplambda}}}}{{\mathrm{D}}^{{\mu }}}$	-
**18**	$\mu$	${\boldsymbol{\lambda}_1}$	${\boldsymbol{\lambda}_2}$	${{\mathbf{I}}^{{{\mathbf{\lambda }}_1}}}{{\mathbf{I}}^{{{\mathbf{\lambda }}_2}}}{{\mathbf{D}}^{\mathbf{\mu }}}$	**Proposed**
When the ${\boldsymbol{\lambda}_2}$ lies within [0.2: 0.5] range, the T controller is obtained.					
19	0	0	T	PT	NA
20	0	1	T	PTI	NA
21	1	0	T	PTD	NA
22	1	1	T	TID	^39^
23	0	${\lambda _1}$	T	${\mathrm{PT}}{{\mathrm{I}}^{{\boldsymbol{\uplambda}}}}$	^50^
24	1	${\lambda _1}$	T	T${{\mathrm{I}}^{{\boldsymbol{\uplambda}}}}{\mathrm{D}}$	^51^
25	$\mu$	0	T	${\mathrm{PT}}{{\mathrm{D}}^{{\mu }}}$	NA
26	$\mu$	1	T	TI${{\mathrm{D}}^{{\mu }}}$	NA
27	$\mu$	${\lambda _1}$	T	T${{\mathrm{I}}^{{\boldsymbol{\uplambda}}}}{{\mathrm{D}}^{{\mu }}}$	^52^

*The parameters of the obtained controllers were rearranged as P, T, I, and D.

## Cuckoo Catfish Optimizer (CCO)

CCO is a novel swarm intelligence meta-heuristic algorithm inspired by the unique parasitic and predatory behaviors of Synodontis multipunctatus (Cuckoo Catfish (CC)) in Lake Tanganyika^53^. Its survival strategy involves learning to seek favorable conditions, cooperatively invading host nests in groups to create chaos, laying its own eggs amid the disturbance, and having its faster-hatching larvae consume the young cichlid. The CCO algorithm mimics these key biological behaviors: the circling search and compressed space strategies simulate its learned movement and cooperative encirclement; the chaotic predation strategy replicates its nest invasion and egg-laying tactic; and the death/regeneration mechanism emulates nature’s survival-of-the-fittest principle through egg predation and renewal^53^. Therefore, CCO is used in several engineering applications^54^. The algorithm mathematically model strategies such as surround search, space compression, chaotic predation, and parasitism to balance global exploration and local exploitation.

- **Inspiration of CCO**.

The CCO algorithm is biologically inspired by the obligate brood parasitism of CC. The primary natural behaviors simulated include the following:

- Surround search and compressed space: CC cooperatively encircle cichlid hosts, constricting the escape space to trap prey.
- Chaotic predation: During nest invasions, catfish create disturbances that allow them to lay eggs undetected among the host brood.
- Death and parasitism: Individuals that fail to find prey face a mortality risk, where new individuals are regenerated to maintain population diversity, thereby simulating natural selection.
- Transition strategy: The population is dynamically split to balance the extensive exploration and intensive exploitation phases.

(b) **Mathematical Formulation of CCO**^53^.

The population is dynamically split to balance the extensive exploration and intensive exploitation phases.

i. **Initialization**.

The positions *x* with *N* population size in a *D*-dimensional search space are initialized as:

11 $$x_{i}^{d}={\mathrm{rand}} \times \left( {U{b^d} - L{b^d}} \right)+L{b^d},i=1, \ldots ,N,d=1, \ldots ,D$$

where $U{b^d}$ and $L{b^d}$ are the upper and lower bounds of the *d*-th dimension and ${\mathrm{rand}}$ is a random number within the [0: 1] range.

ii. **Compressed space strategy**.

**Fig. 6:**
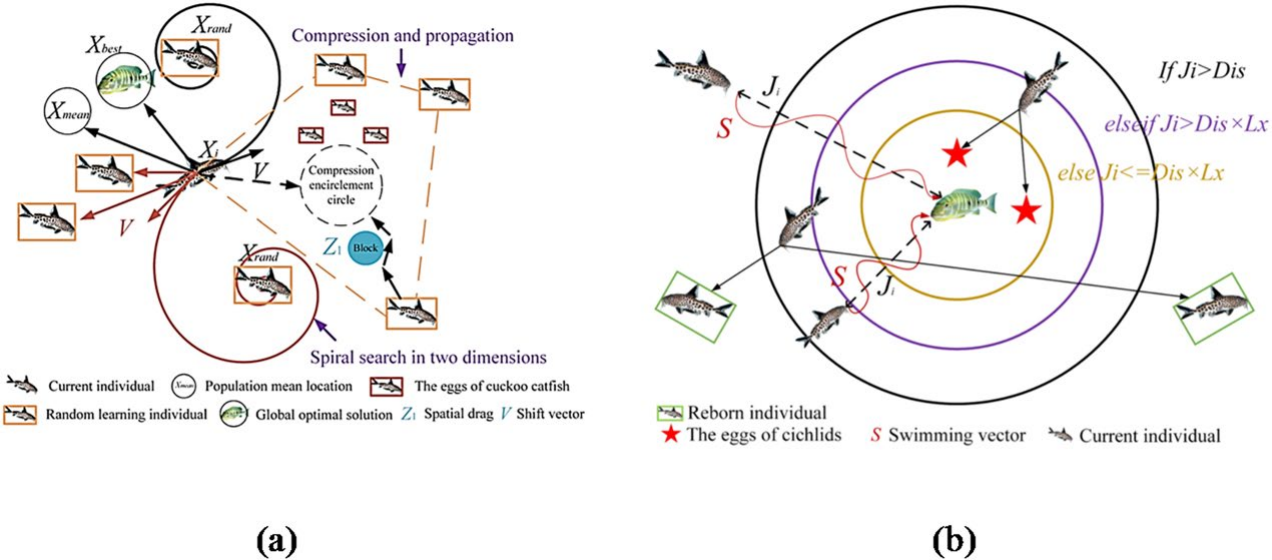
(a) The compression of space behavior and surround search behavior of CC; (b) The predation behavior of CC.

To facilitate prey capture, a population of cuckoo catfish cooperates to constrict the search area. This process gradually narrows the range of the prey and reduces its escape options, as shown in Fig. 6(a).This strategy simulates cooperative space reduction:

12 $$X_{i}^{{{\mathrm{new}}}}={X_i}+{Z_1} \times \mid rd\mid \times \left( {\frac{{{X_{{\mathrm{best}}}}+{X_{{r_1}}}}}{2}{X_{{r_2}}}} \right)+\frac{{{r_3}}}{2} \times \left( {{X_{{r_3}}} - {X_{{r_4}}}} \right)$$

13 $$X_{i}^{{{\mathrm{new}}}}={Z_2} \times \left( {{X_{{i_1}}}+\mid rd\mid \times \left( {{X_{{i_1}}} - {X_{{i_2}}}} \right)} \right)+\left( {1 - {Z_2}} \right) \times {X_i}$$

14 $$X_{i}^{{{\mathrm{new}}}}={X_{{r_1}}}+\mid rd\mid \times \left( {{X_{{\mathrm{best}}}} - {X_i}+{X_{{r_2}}} - {X_{{r_3}}}} \right)$$

where $X_{i}^{{{\mathrm{new}}}}$ is the updated position of the ${i^{th}}$ individual, ${Z_1},{Z_2} \in \left\{ {0,1} \right\}$ simulate environmental resistance, $rd\sim \mathcal{N}\left( {0,1} \right)$, C is a decreasing factor, ${X_{\mathrm{r}}}$ is a random individual, and ${X_{{\mathrm{best}}}}$ is the global best position.

iii. **Surround Search Strategy**.

- When the position of the selected random individual ${X_r}$ is better than the position of ${X_i}$, ${X_i}~$will approach ${X_r}$ in a spiral search pattern to ensure that Xi explores a broader space.

15 $$\begin{array}{*{20}{c}} {}&{X_{i}^{{{\mathrm{new}}}}=\left\{ {\begin{array}{*{20}{c}} {{X_e}+F \times {R_1} \times \frac{{{\mathrm{step}}}}{T}+{T^n} \times s \times \left( {1 - {R_1}} \right) \times \mid {\mathrm{step}}\mid +V \times \frac{t}{T},}&{{\mathrm{if~mod}}\left( {i,2} \right)=0} \\ {{X_e}+F \times {R_1} \times \frac{{{\mathrm{step}}}}{T}+{T^n} \times c \times \left( {1 - {R_1}} \right) \times \mid {\mathrm{step}}\mid +V \times \frac{t}{T},}&{{\mathrm{otherwise}}} \end{array}} \right.} \end{array}$$

where $$T={\left( {1 - {\mathrm{sin}}\left( {\frac{\pi }{2}{{ \times }}\frac{{It}}{{MaxIt}}} \right)} \right)^{\left( {It/MaxIt} \right)}}$$, $$c=a{e^{\left( {b - \theta } \right)/2}}{\mathrm{cos}}\left( \theta \right)$$, $$s=a{e^{\left( {b - \theta } \right)/2}}{\mathrm{sin}}\left( \theta \right)$$, $$\theta =\left( {1 - 10i/N} \right)\pi$$, and $$a=1.34,b=0.3.$$

b) When the fitness value of the new position ${X_i}^{{new}}~$is better than that of the previous one ${X_i}$, the underperforming individuals in the population will observe the surrounding behavior of ${X_i}$ and be guided to the position of ${X_i}$, position with a 25% probability. The search method is performed using a spherical mode:

16 $$\begin{array}{*{20}{c}} {}&{X_{i}^{{{\mathrm{new}}}}=\left\{ {\begin{array}{*{20}{c}} {{\mathrm{Rot~}}{{\mathrm{X}}_r}+2wF\cos \left( {R{t_1}} \right)\sin \left( {R{t_2}} \right)\left( {{\mathrm{Rot~}}{{\mathrm{X}}_r} - X_{i}^{{{\mathrm{new}}}}} \right),}&{{X_q}=1} \\ {{\mathrm{Rot~}}{{\mathrm{X}}_r}+2wF\sin \left( {R{t_1}} \right)\cos \left( {R{t_2}} \right)\left( {{\mathrm{Rot~}}{{\mathrm{X}}_r} - X_{i}^{{{\mathrm{new}}}}} \right),}&{{X_q}=2} \\ {{\mathrm{Rot~}}{{\mathrm{X}}_r}+2wF\cos \left( {R{t_2}} \right)\left( {{\mathrm{Rot~}}{{\mathrm{X}}_r} - X_{i}^{{{\mathrm{new}}}}} \right),~~~~~~~~~~~~~~~~}&{{X_q}=3} \end{array}} \right.} \end{array}$$

$${\mathrm{with}}~w=1 - \frac{{{e^{\frac{{wt}}{{wt+n}}}} - 1}}{{e - 1}},~{\mathrm{and}}~{\mathrm{Rot~X}}=\left[ {{X_{{\mathrm{best}}}},~{X_{{\mathrm{best2}}}},~{X_{{\mathrm{best3}}}},~{\mathrm{mean}}\left( X \right)} \right].$$

iv. **Transition strategy**

To balance exploration and exploitation, the following transition is used:

17 $$\begin{array}{*{20}{c}} {}&{X_{i}^{{{\mathrm{new}}}}=\left\{ {\begin{array}{*{20}{c}} {\frac{C}{2}\left( {{r_1}{X_{{\mathrm{best}}}}{r_3}{X_i}} \right)+{T^2} \times {\mathrm{lev}}\left( D \right) \times \mid {\mathrm{Ste}}{{\mathrm{p}}_2}\mid ,}&{{\mathrm{if~mod}}\left( {i,2} \right)=0} \\ {\frac{{{X_{{\mathrm{best}}}}+{X_i}}}{2}+De\left( {2{R_1}{\mathrm{Ste}}{{\mathrm{p}}_2} - \frac{{2\mu }}{2}\left( {De{R_3} - 1} \right)} \right),}&{{\mathrm{otherwise}}} \end{array}} \right.} \end{array}$$

where $De=C \times F$ and ${\mathrm{lev}}\left( D \right)$ is the D-dimensional Lévy distribution.

v. **Chaotic predation strategy**

Models the host’s chaotic response during invasion:

18 $$\begin{array}{*{20}{c}} {}&{X_{i}^{{{\mathrm{new}}}}=\left\{ {\begin{array}{*{20}{c}} {{X_{{\mathrm{best}}}}+F \times S \times \left( {{X_{{\mathrm{best}}}} - {X_i}} \right),}&{{J_i}>{J_{{\mathrm{in}}}}} \\ {{X_{{\mathrm{best}}}}\left( {1{\mathrm{+}}{T^5}{C_Y}E} \right)+FS\left( {{X_{{\mathrm{best}}}} - {X_i}} \right),}&{{J_{{\mathrm{in}}}} \times Lx<{J_i} \leqslant {J_{{\mathrm{in}}}}} \\ {{X_{{\mathrm{best}}}}\left( {1{\mathrm{+}}{T^5}{G_S}} \right)+FS\left( {{X_{{\mathrm{best}}}} - {X_i}} \right),}&{{J_i} \leqslant {J_{{\mathrm{in}}}} \times Lx} \end{array}} \right.} \end{array}$$

where ${J_i}=\frac{1}{D}\mathop \sum \limits_{{j=1}}^{D} \frac{{{X_i} - {X_{{\mathrm{best}}}}}}{{{X_{{\mathrm{worst}}}} - {X_{{\mathrm{best}}}}}}$, ${J_{{\mathrm{in}}}}$ is the initial average aggregation, ${C_Y}$ is a chaotic coefficient, and ${G_S}\sim \mathcal{N}\left( {0,{C^2}} \right)$^53^.

vi. **Death and parasitism strategy**

The CCO algorithm simulates mortality and regeneration using the following update rule:

19 $$\begin{array}{*{20}{c}} {}&{X_{i}^{{{\mathrm{new}}}}=\left\{ {\begin{array}{*{20}{c}} {{r_1}\left( {U{p_c} - Lo{w_c}} \right)+Lo{w_c},}&{{\mathrm{if~rand}}>C} \\ {{r_1}\left( {Ub - Lb} \right)+Lb,}&{{\mathrm{otherwise}}} \end{array}} \right.} \end{array}$$

$${\mathrm{where~}}U{p_c}={\mathrm{max}}\left( {{\mathrm{best}}} \right),{\mathrm{~}}Lo{w_c}={\mathrm{min}}\left( {{\mathrm{best}}} \right),{\mathrm{~and~best}}={X_{{\mathrm{best}}}} \times \left( {{\mathrm{lev}}\left( 1 \right) \times A+\mid r{d_1}\mid \times \left( {1 - A} \right)} \right).$$

The position of each individual will be updated according to the following rule:

20 $$\begin{array}{*{20}{c}} {}&{{X_i}=\left\{ {\begin{array}{*{20}{c}} {X_{i}^{{{\mathrm{new}}}},}&{F_{i}^{{{\mathrm{new}}}}<{F_i}} \\ {{X_i},}&{{\mathrm{otherwise}}} \end{array}} \right.} \end{array}$$

**Fig. 7:**
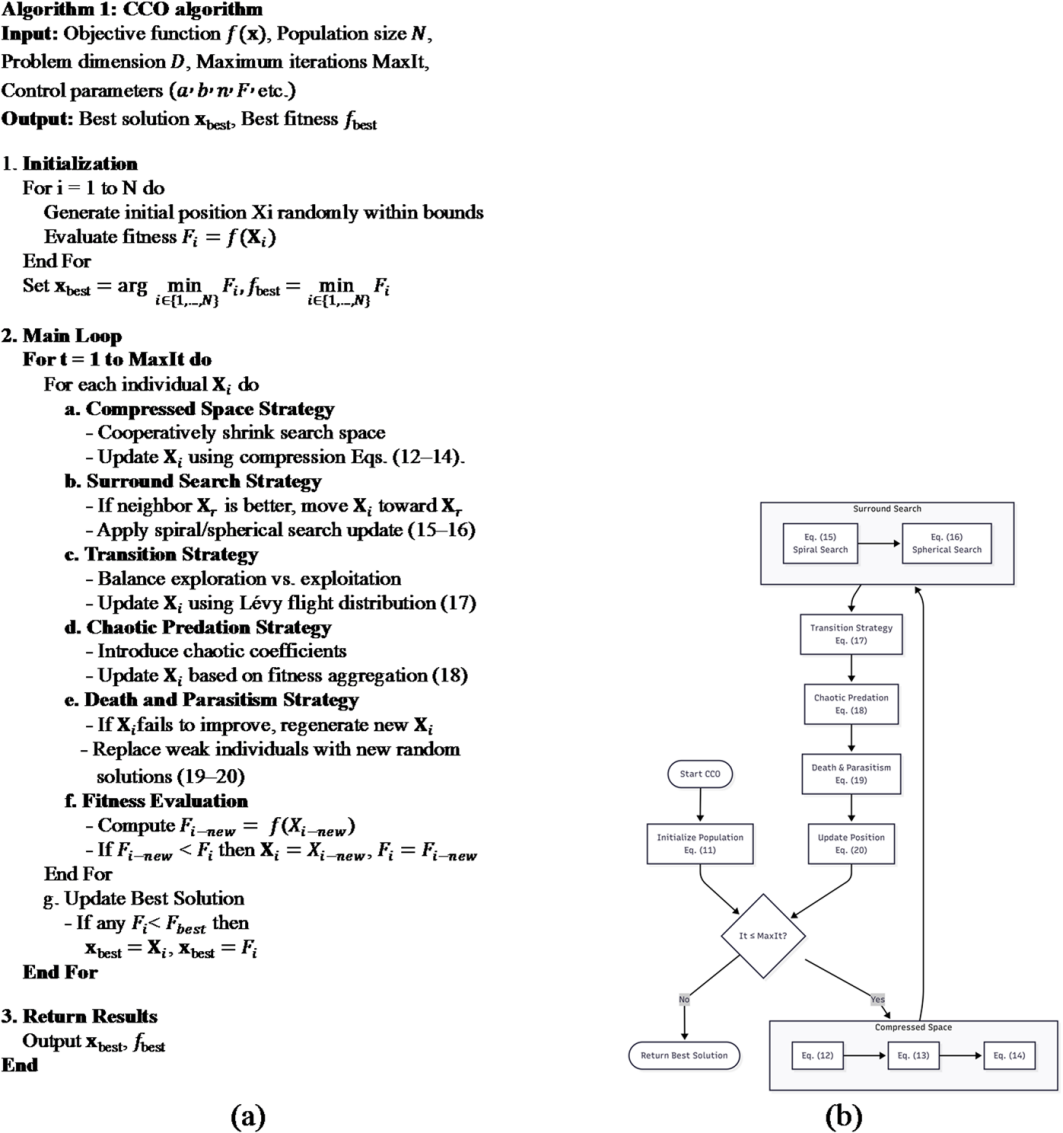
The CCO algorithm: (a) Pseudocode and (b) Flowchart.

The key parameters are: $It$: Current iteration number, *T*: Shrinkage factor, $MaxIt$: Maximum number of iterations, ${J_i},{J_{{\mathrm{in}}}}$: Aggregation degree metrics, ${\mathrm{lev}}\left( D \right)$: Lévy flight distribution, $S,~F,Z,~W$: Behavioral control parameters within the [0: 1] range. $RotX$: Candidate sphere center positions, $Rt$: Rotation angle (range [0, 2π]). Table 2 presents the remaining key parameters of CCO and their recommended values^53^. The pseudocode and flowchart of the proposed CCO algorithm are illustrated in Figs. 7(a) and 7(b), respectively. A complete description of the CCO algorithm, including its mathematical formulation and implementation details, is available in^53^.

**Table 2:** CCO parameter definitions.

Category	Parameter	Symbol	Role	Recommended value
Population	Population size	*N*	Number of individuals	50
	Problem dimension	*D*	Depends on the problem	6 for two area IPS 18 for three area IPS
Exploration	Spiral constants	$a,b$	Control spiral shape and density	$a=1.34,b=0.3$
	Shrinkage exponent	*n*	Controls contraction rate	2
	Direction factor	*F*	Random movement direction	-1 or 1 (random)
Exploitation	Decreasing factor	*C*	Balances exploration vs. exploitation	$C=1 - It/MaxIt$
	Death probability	$die$	Probability of individual removal	0.02
Chaotic search	Lévy exponent	$\beta$	Step length in Lévy flights	1.5
Miscellaneous	Kinetic energy	*E*	Decreases with iteration	$E=T+{r_1}$

Given the focus on the FOIID controller, CCO was selected as the optimizer due to its proven ability to balance exploration and exploitation in multimodal, nonlinear optimization landscapes. This ensures a robust performance evaluation of the FOIID controller without diverting focus to optimizer complexity.

### Integration of CCO with FOIID Controller based ITAE criterion

Control system performance is typically evaluated using established error-minimization metrics, including mean square error (MSE), integral of squared error (ISE), integral of absolute error (IAE), integral of time-weighted absolute error (ITAE), and integral of time-weighted squared error (ITSE). The ITAE criterion, which integrates the absolute error multiplied by time, is particularly prevalent in LFC applications^1^, due to its emphasis on persistent errors and alignment with frequency regulation requirements. Consequently, this study employs the ITAE metric for comparative analysis, leveraging its demonstrated efficacy in balancing responsiveness and stability for complex control systems.

**Fig. 8:**
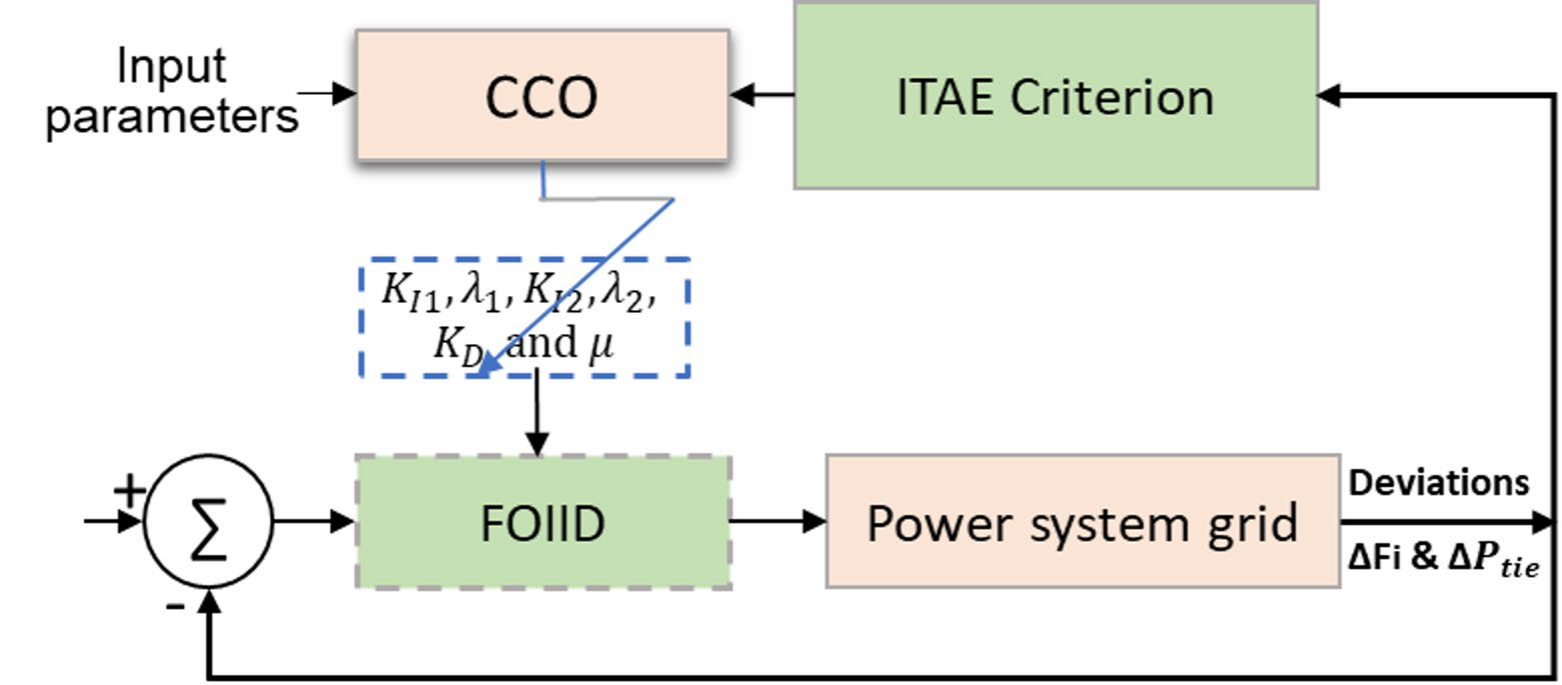
System design-based CCO-FOIID based ITAE.

The parameters of the FOIID controller were optimized using the CCO based on the ITAE criterion within a closed-loop system as shown in Fig. 8. The CCO algorithm iteratively adjusted the six controller gains, ${\lambda _1},{\lambda _2},\mu ,{K_{I1}},{K_{I2}},{\mathrm{and}}~{K_p},$ to minimize the ITAE cost function. The PS outputs, ${{\boldsymbol{\Delta}}}{f_i}$ and ${{\boldsymbol{\Delta}}}{P_{{\mathrm{tie}}}}$, were used to compute the cost function. Guided by its bio-inspired hunting strategies, the CCO algorithm progressively refined the parameter sets over multiple iterations to identify the optimal configuration. The optimization process was repeated across multiple independent runs to ensure consistency and robustness of the results.

For two-area and three-area systems, the ITAE cost functions are expressed as:

$${\mathrm{ITAE}}{{\mathrm{~}}_{{\mathrm{two}} - {\mathrm{area}}}}{\mathrm{~}}=\mathop \smallint \limits_{0}^{{{{\mathrm{t}}_{{\mathrm{sim}}}}}} t \cdot \left( {{{\boldsymbol{\Delta}}}{{\mathrm{f}}_1}+{{\boldsymbol{\Delta}}}{{\mathrm{f}}_2}+{{\boldsymbol{\Delta}}}{{\mathrm{P}}_{{\mathrm{tie-line}}}}} \right){\mathrm{dt}}$$

21 $${\mathrm{ITAE}}{{\mathrm{~}}_{{\mathrm{three}} - {\mathrm{area}}}}=\mathop \smallint \limits_{0}^{{{{\mathrm{t}}_{{\mathrm{sim}}}}}} t \cdot \left( {{{\boldsymbol{\Delta}}}{{\mathrm{f}}_1}+{{\boldsymbol{\Delta}}}{{\mathrm{f}}_2}+{{\boldsymbol{\Delta}}}{{\mathrm{f}}_3}+{{\boldsymbol{\Delta}}}{{\mathrm{P}}_{{\mathrm{tie1}}}}+{{\boldsymbol{\Delta}}}{{\mathrm{P}}_{{\mathrm{tie2}}}}+{{\boldsymbol{\Delta}}}{{\mathrm{P}}_{{\mathrm{tie3}}}}} \right){\mathrm{dt~}}$$

where ${{\mathrm{t}}_{{\mathrm{sim}}}}$ represents the simulation duration. The optimization objective is to minimize the ITAE value, subject to the following parameter constraints:

$${\mathrm{~K}}_{{{{\mathrm{I}}_{1,2}}}}^{{{\mathrm{LB}}}} \leqslant {{\mathrm{K}}_{{\mathrm{I}}1}},{\mathrm{~}}{{\mathrm{K}}_{{\mathrm{I}}2}} \leqslant {\mathrm{K}}_{{{{\mathrm{I}}_{1,2}}}}^{{{\mathrm{UB}}}},{\mathrm{~~}}$$

$$~~{\mathrm{K}}_{\lambda }^{{{\mathrm{LB}}}} \leqslant {\lambda _1},{\lambda _2} \leqslant {\mathrm{K}}_{\lambda }^{{{\mathrm{UB}}}},$$

22 $$~{\mathrm{K}}_{{\mathrm{D}}}^{{{\mathrm{LB}}}} \leqslant {{\mathrm{K}}_{\mathrm{D}}} \leqslant {\mathrm{K}}_{{\mathrm{D}}}^{{{\mathrm{UB}}}},$$

$${\mathrm{~K}}_{\mu }^{{{\mathrm{LB}}}} \leqslant \mu \leqslant {\mathrm{K}}_{\mu }^{{{\mathrm{UB}}}}$$

The superscripts $LB$ and $UB$ denote the lower and upper bounds of each parameter.

Population size is critical for real-time implementation of CCO-optimized FOIID systems^45^. According to^55^, smaller populations can yield satisfactory results while being more computationally efficient for real-time LFC applications. The FOIID parameters were constrained to specific ranges: gains for two-area non-reheat systems were bounded within [0, 3], while three-area systems used were bounded within the [0, 2], with fractional orders ${\lambda _{1,}},~{\lambda _2}$ and $\mu$ limited to [0, 1.5] as per^1,20,23^.

### The rationale for selecting CCO Algorithm

The choice of the CCO as the tuning algorithm was motivated by several technical and practical considerations:

1. The “No Free Lunch” theorem: This theorem justifies exploring new optimizers for LFC applications, as no single algorithm is universally optimal for all optimization problems.
2. Biological inspiration and novelty: CCO balances global exploration and local exploitation through its multi-strategy design, including space compression, chaotic predation, and Lévy flights. These mechanisms provide a natural balance between searching broadly for global optima and refining solutions locally, which is critical in complex optimization landscapes such as those found in LFC.
3. Avoidance of premature convergence: The chaotic predation and regeneration strategies maintain population diversity, thereby reducing the risk of premature convergence and ensuring a more reliable global search capability.
4. Performance superiority under comparable conditions: As demonstrated in the studied test systems (Sect.  4.3), CCO consistently outperformed established optimizers such as HHO and GWO under identical iteration and population settings. This superiority was evident in lower ITAE values, faster settling times, and reduced overshoot, confirming its suitability for LFC applications.
5. Scalability to high-dimensional problems: The FOIID controller introduces multiple fractional-order parameters, rendering the optimization problem high-dimensional and requiring a robust search mechanism.

In summary, CCO serves as a rigorous, high-performance, and unbiased computational tool. Its proven capabilities ensure that the FOIID controller is tuned under optimal conditions, thereby enabling a fair, conclusive, and focused evaluation of its intrinsic merits and contributions to control system design.

## Results and discussion

This study evaluates two LFC models: a linear two-area system with non-reheat thermal units, as shown in Fig. 1(c), and a nonlinear three-area IPS integrating GRC dynamics with thermal-thermal-hydro units, depicted in Fig. 2. The former validates the efficacy of the CCO algorithm and the proposed FOIID controller in addressing LFC challenges. Using MATLAB/Simulink, the FOIID controller gains were optimized via CCO over 30 independent runs, with 50 iterations and a population size of 50. For clarity, although the simulation durations were set to 10 s for the two-area system and 200 s for the three-area system, the figures are truncated to 7 s and 150 s, respectively, to enhance the visualization of critical transient dynamics.

The FOIID controller exhibited superior performance compared to PID, FOPID, and CCs in mitigating frequency deviations and tie-line power errors, particularly under load variations, GRC nonlinearities, and multi-area interactions. Key performance indices, including reduced settling time, minimized undershoot/overshoot, negligible steady-state error, and improved ITAE values, collectively demonstrate its efficacy. Implemented in MATLAB/Simulink, this framework highlights the FOIID controller’s robustness in addressing multi-modal LFC challenges.

### Linear two area model

This study first evaluates the two-area non-reheat thermal PS using a CCO-optimized FOPID controller, benchmarking its performance against the published DSA-FOPID method^20^ to establish the efficacy of CCO in addressing LFC challenges. Subsequently, the proposed CCO-optimized FOIID controller is implemented to demonstrate enhanced system stability. For a fair comparison, both the FOPID and FOIID controllers are evaluated under the ITAE criterion. The optimized parameters for all controller configurations under a 10% SLP in Area 1 are detailed in Table 3.

**Table 3:** Optimized controller parameters of two-area non-thermal PS at 10% SLC at area 1.

Algorithm	ISFS: PID^56^	DSA: FOPID^20^	CCO: FOPID	CCO: FOIID
Controller parameters	${K_P}=1.6293$	${K_P}=~2.0331$	${K_P}=~~2.0396$	${K_{I1}}=3.0000$
	${K_I}=2.0000$	${K_I}=2.9999$	${K_I}=3.0000$	${\lambda _1}=1.1017$
	${K_D}=0.5882$	${K_D}=0.4670$	${K_D}=0.4305$	${K_{I2}}=3.0000$
		$\lambda =1.0007$	$\lambda =1.00108$	${\lambda _2}=0.5635$
		$\mu =~1.0517$	$\mu =~1.0809$	${K_D}=1.5122$
				$$\mu =0.6206$$

**Table 4:** Performance analysis of two-area model at 10% SLC.

Algorithm	Objective functions	Settling time T_s_ (s)			Undershoot(-ve)		
	ITAE	$\Delta {F_1}$	$\Delta {F_2}$	$\Delta {P_{tie}}$	$\Delta {F_1}$	$\Delta {F_2}$	${P_{tie}}$
ISFS: PID^56^	n/a	2.15	3.66	3.01	0.0818	0.0423	0.01555
DSA: FOPID^20^	0.0778	1.13	2.73	2.23	0.0822	0.0416	0.01485
CCO: FOPID	0.07683	1.13	2.73	2.22	0.0839	0.0427	0.01510
CCO: FOIID	0.05165	2.3	1.83	1.65	0.0850	0.03922	0.01298

A comprehensive performance analysis, summarized in Table 4, evaluates CCO-FOPID and CCO-FOIID under the ITAE criterion against established schemes including ISFS-PID^56^ and DSA: FOPID^20^. The fractional-order integrators ${\lambda _1}\left( {1.1017} \right){\mathrm{~}}$ and $~{\lambda _2}$ (0.5635) provide continuous tuning of the memory characteristics of the integral actions. Smaller values increase the influence of recent errors and therefore improve transient responsiveness, whereas larger values distribute the memory over a longer error history, which can enhance low-frequency error compensation and steady-state regulation.

**Fig. 9:**
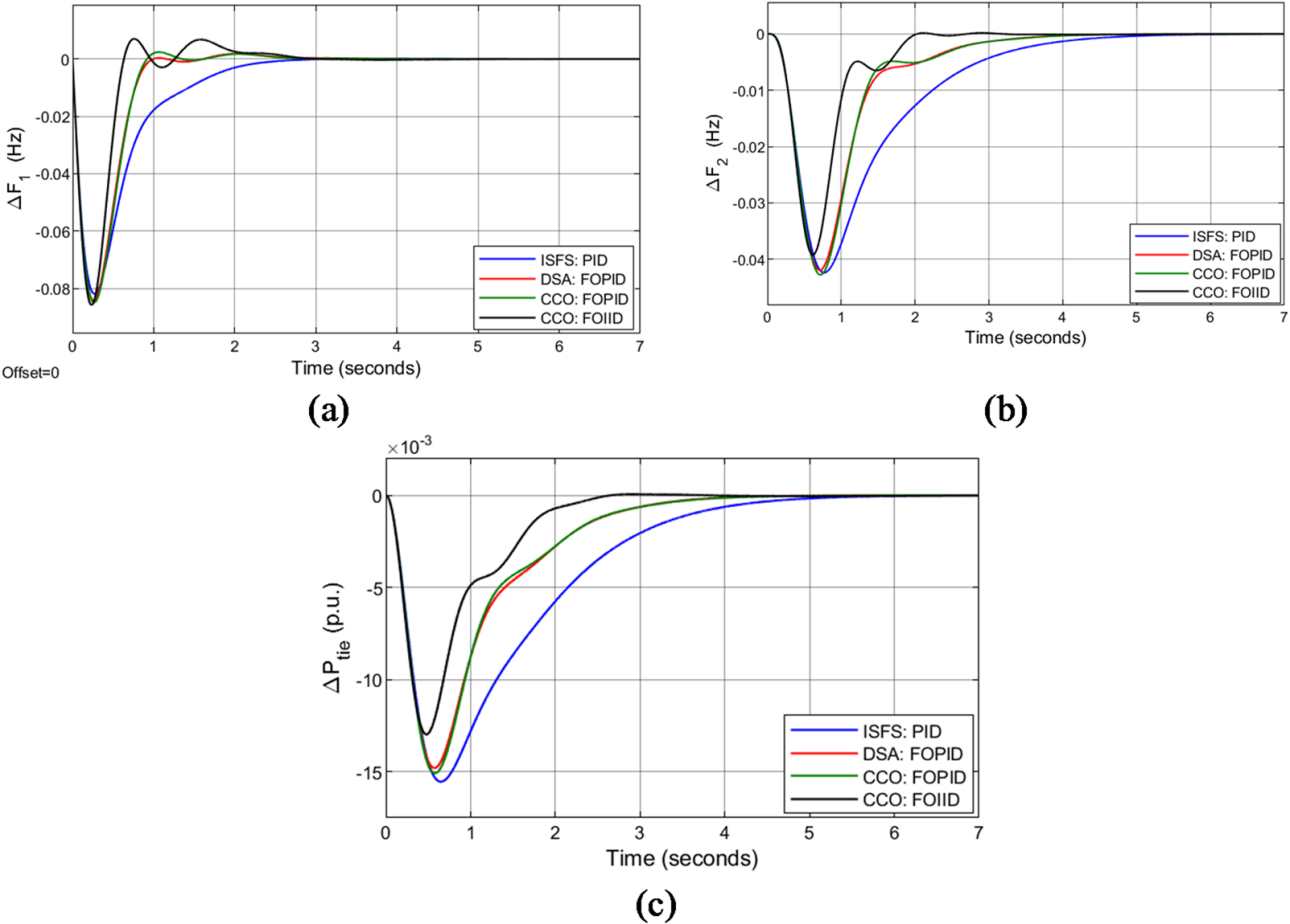
Dynamic response comparison of controllers for two area model at ΔP_D1_ = 0.1 puMW (a) $\Delta {F_1},$ (b) $\Delta {F_2},$ and (c) $\Delta {P_{tie}}$.

The CCO-FOPID controller achieves a marginally lower ITAE (0.07684) than DSA-FOPID (0.0778), confirming CCO’s suitability for LFC applications. The CCO-FOIID controller demonstrates a more substantial improvement, achieving a 33% reduction in ITAE (0.05165) alongside enhanced settling times and reduced undershoots in frequency and tie-line power deviations. Figure 9 illustrates the corresponding dynamic responses of ${{\boldsymbol{\Delta}}}{F_1}$ and ${{\boldsymbol{\Delta}}}{F_2}$ and tie-line power flow $\left( {{{\boldsymbol{\Delta}}}{P_{tie}}} \right)$ under 10% SLP.

**Fig. 10:**
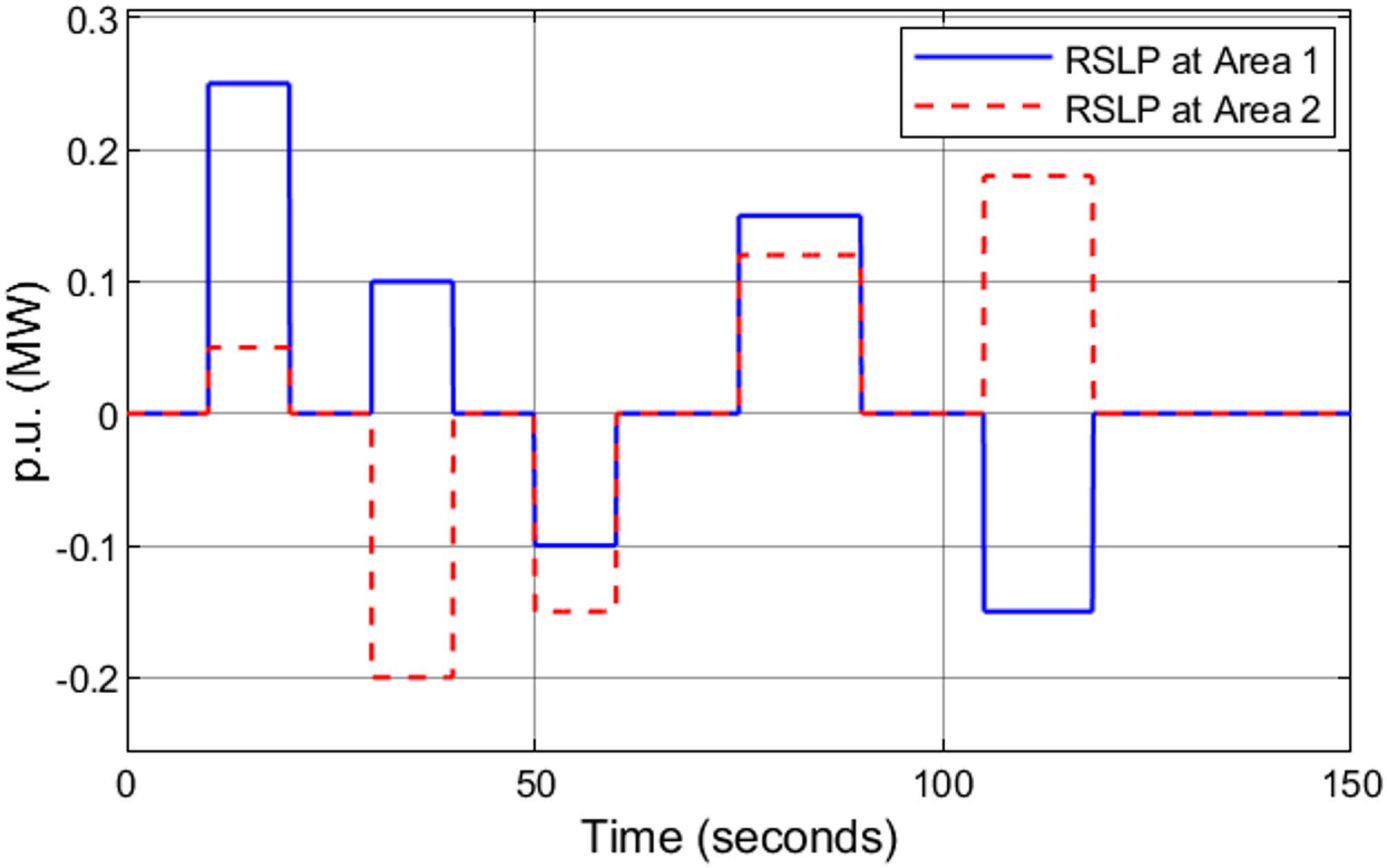
Random step load pattern applied at area-1 and area-2 simultaneously.

**Fig. 11:**
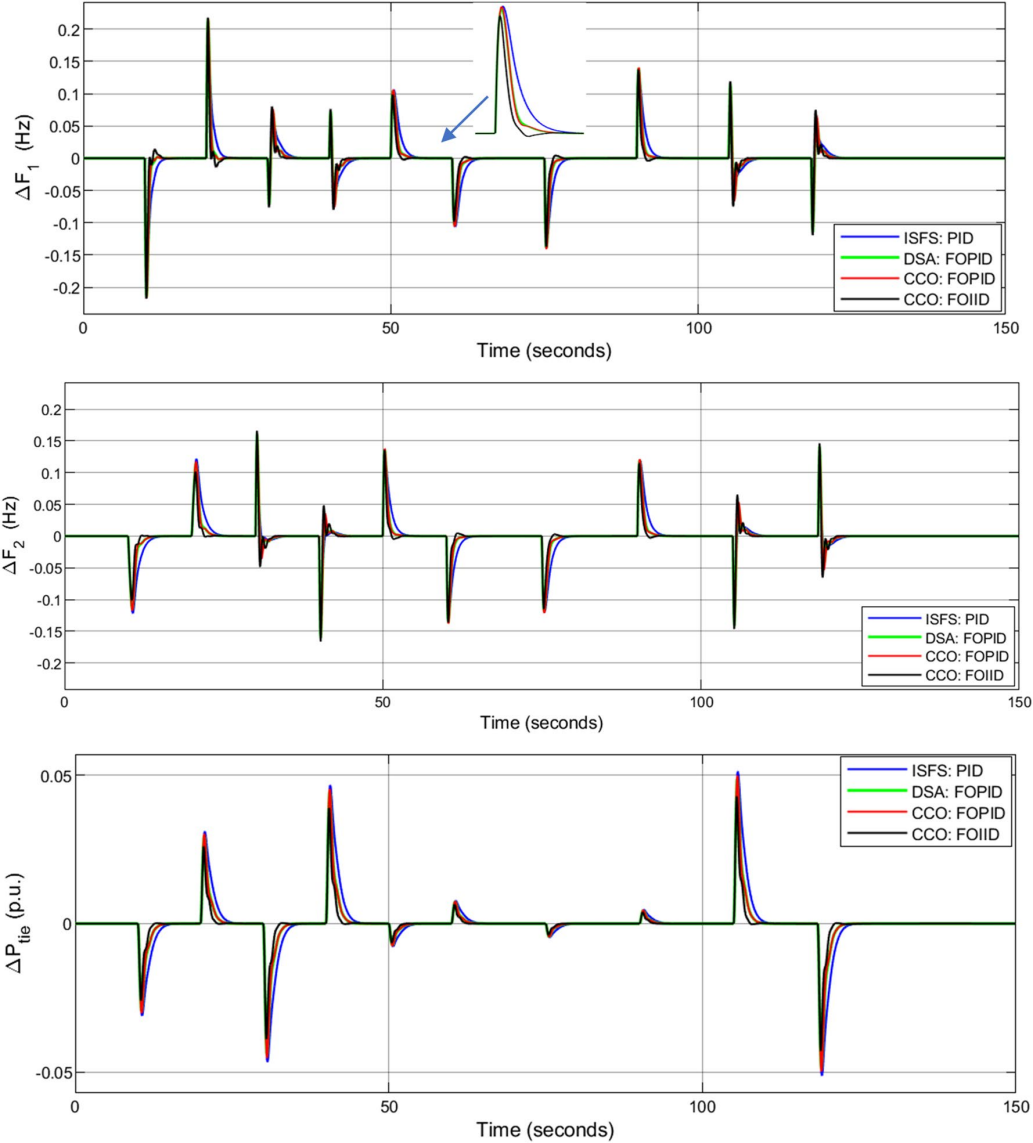
Robustness analysis for two area model under RSLP.

Furthermore, the proposed controllers, along with the other benchmark controllers, are further evaluated under a random SLP (RSLP) in both area 1 and area 2, as depicted in Fig. 10. The transient responses, shown in Fig. 11, confirm the superiority and validate the stability of the two-area model under the CCO: FOPID and FOIID controllers.

### Three area model with GRC nonlinearity

This study investigates a three-area IPS incorporating hydrothermal generation units and GRC nonlinearities. To evaluate system performance, a 1% SLP is simultaneously applied to all three areas at $t~=~0$ s. The CCO algorithm is employed to tune the PID, FOPID, and the proposed FOIID controllers. The performance of these controllers is benchmarked against several established methods from literature, including hBFOA-PSO adjusted PI controller^57^, ISFS adjusted PID controller^56^, DSA adjusted FOPID and FOPI-FOPD controllers^20^, as well as the recently published WHO adjusted PI-(1 + FOPID) controller^23^ and optimized CGO FOPID-FOPI controller^1^. The optimized parameters for all controllers are comprehensively summarized in Table 5, providing a complete reference for tuning comparisons and methodological validation.

**Table 5:** Optimized controller parameters under 1% SLP at each area.

Algorithm	ISFS: PID^56^	DSA: FOPID ^20^	DSA: FOPI- FOPD ^20^	WHO: PI- (1 + FOPID)^23^	CGO: FOPID- FOPI^1^	CCO: FOIID
Controller parameters	**Area 1: Thermal**	**Area 1: Thermal**	**Area 1: Thermal**	**Area 1: Thermal**	**Area 1: Thermal**	**Area 1: Thermal**
	${K_P}=1.1539e - 12$	${K_P}=0.0001$	${K_{P1}}=0.0316$	${K_{P1}}=2.7235$	${K_{P1}}=1.9248$	${K_{I1}}=0.2487$
	${K_I}=0.0577$	${K_I}=0.1253$	${K_{P2}}=0.3346$	${K_{P2}}=0.0069$	${K_{P2}}=1.4875$	${\lambda _1}=0.0202$
	${K_D}=0.1606$	${K_D}=0.1206$	${K_I}=0.3424$	${K_{I1}}=0.56516$	${K_{I1}}=0.3518$	${K_{I2}}=0.2015$
	**Area 2: Thermal**	$\lambda =1.0000$	${K_D}=0.0271$	${K_{I2}}=0.1676$	${K_{I2}}=1e - 12$	${\lambda _2}=1.0123$
	${K_P}=1.1539e - 12$	$\mu =0.5004$	$\lambda =1.0000$	${K_D}=3.0000$	${K_D}=1.8881$	${K_D}=1.0000$
	${K_I}=0.0577$	**Area 2: Thermal**	$\mu =0.6354$	$\lambda =1.213e - 9$	${\lambda _1}=1.0000$	$\mu =0.8523$
	${K_D}=0.1606$	${K_P}=0.0001$	**Area 2: Thermal**	$\mu =0.8723$	${\lambda _2}=0.0930$	**Area 2: Thermal**
	**Area 3: Hydro**	${K_I}=0.1253$	${K_{P1}}=0.0316$	**Area 2: Thermal**	$\mu =1.0000$	${K_{I1}}=0.9397$
	${K_P}=1.1539e - 12$	${K_D}=0.1206$	${K_{P2}}=0.3346$	${K_{P1}}=2.7235$	**Area 2: Thermal**	${\lambda _1}=0.0897$
	${K_I}=0.0577$	$\lambda =1.0000$	${K_I}=0.3424$	${K_{P2}}=0.0069$	${K_{P1}}=0.9875$	${K_{I2}}=0.1911$
	${K_D}=0.1606$	$\mu =0.5004$	${K_D}=0.0271$	${K_{I1}}=0.56516$	${K_{P2}}=1.7e - 7$	${\lambda _2}=1.0089$
		**Area 3: Hydro**	$\lambda =1.0000$	${K_{I2}}=0.1676$	${K_{I1}}=0.2773$	${K_D}=2.0000$
		${K_P}=0.0001$	$\mu =0.6354$	${K_D}=3.0000$	${K_{I2}}=1.3016$	$\mu =1.0052$
		${K_I}=0.0264$	**Area 3: Hydro**	$\lambda =1.213e - 9$	${K_D}=1.9500$	**Area 3: Hydro**
		${K_D}=0.0350$	${K_{P1}}=0.0316$	$\mu =0.8723$	${\lambda _1}=1.0000$	${K_{I1}}=0.0000$
		$\lambda =1.0000$	${K_{P2}}=0.3346$	**Area 3: Hydro**	${\lambda _2}=1e - 5$	${\lambda _1}=0.1937$
		$\mu =0.7376$	${K_I}=0.3424$	${K_{P1}}=2.547e - 5$	$\mu =1.0000$	${K_{I2}}=0.0083$
			${K_D}=0.0271$	${K_{P2}}=5.2797e - 11$	**Area 3: Hydro**	${\lambda _2}=1.4999$
			$\lambda =1.0000$	${K_{I1}}=0.01069$	${K_{P1}}=0.0020$	${K_D}=0.1278$
			$\mu =0.6354$	${K_{I2}}=0.47815$	${K_{P2}}=0.5422$	$\mu =1.03$01
				${K_D}=0.0000$	${K_{I1}}=0.0305$	
				$\lambda =0.6126$	${K_{I2}}=0.4657$	
				$\mu =0.0000$	${K_D}=0.1605$	
					$${\lambda _1}=1.000$$	
					$${\lambda _2}=0.1376$$	
					$$\mu =1.0000$$	

**Table 6:** Performance analysis of differnet schemes at 1% SLP for each area based on ITAE criterion.

Cost functions		hBFOA- PSO: PI^57^	ISFS: PID^56^	DSA: FOPID ^20^	DSA: FOPI - FOPD^20^	WHO: PI (1 + FOPID) ^23^	CGO: FOPID -FOPI ^1^	CCO: FOIID
ITAE		n/a	n/a	156.32	147.56	132.75	82.67	74.26
Signal statistics								
$\Delta {F_1}$	Ts (s) (2% band)	171.1	107.6	137.5	90.3	82.2	60.59	41.1
	OS+ *e-2	6.060	4.906	5.822	5.825	5.318	3.678	3.012
	US- *e-1	1.814	1.421	1.753	1.797	1.792	1.365	1.478
$\Delta {F_2}$	Settling time	171.1	107.6	137.5	90.3	82.2	60.59	44.55
	OS+ *e-2	6.060	4.906	5.822	5.825	5.317	3.738	2.970
	US- *e-1	1.814	1.421	1.753	1.797	1.791	1.366	1.477
ΔF_3_	Settling time	171.1	107.6	137.6	90.0	81.6	58.71	38.0
	OS+ *e-2	5.721	4.564	5.307	5.678	5.019	3.384	2.669
	US- *e-1	1.821	1.378	1.770	1.829	1.822	1.327	1.437
$\Delta {P_{tie1}}$	Settling time	119.1	65.28	109.1	68.7	76.2	77.43	30.8
	OS+ *e-4	20.10	1.552	19.690	21.620	21.890	7.434	12.762
	US- *e-2	1.480	1.431	1.515	1.499	1.574	1.310	1.297
$\Delta {P_{tie2}}$	Settling time	119.1	65.28	109.1	68.7	76.2	77.43	43.0
	OS+ *e-4	20.10	1.552	19.690	21.620	21.82	10.050	1.815
	US- *e-2	1.472	1.431	1.515	1.499	1.572	1.323	1.363
$\Delta {P_{tie3}}$	Settling time	139.7	94.83	130.9	74.7	95.2	62.47	40.0
	OS+ *e-2	2.962	2.862	3.031	2.998	3.134	2.619	2.622
	US- *e-3	0.411	0.3103	3.939	4.325	4.355	1.678	0.6285

**Fig. 12:**
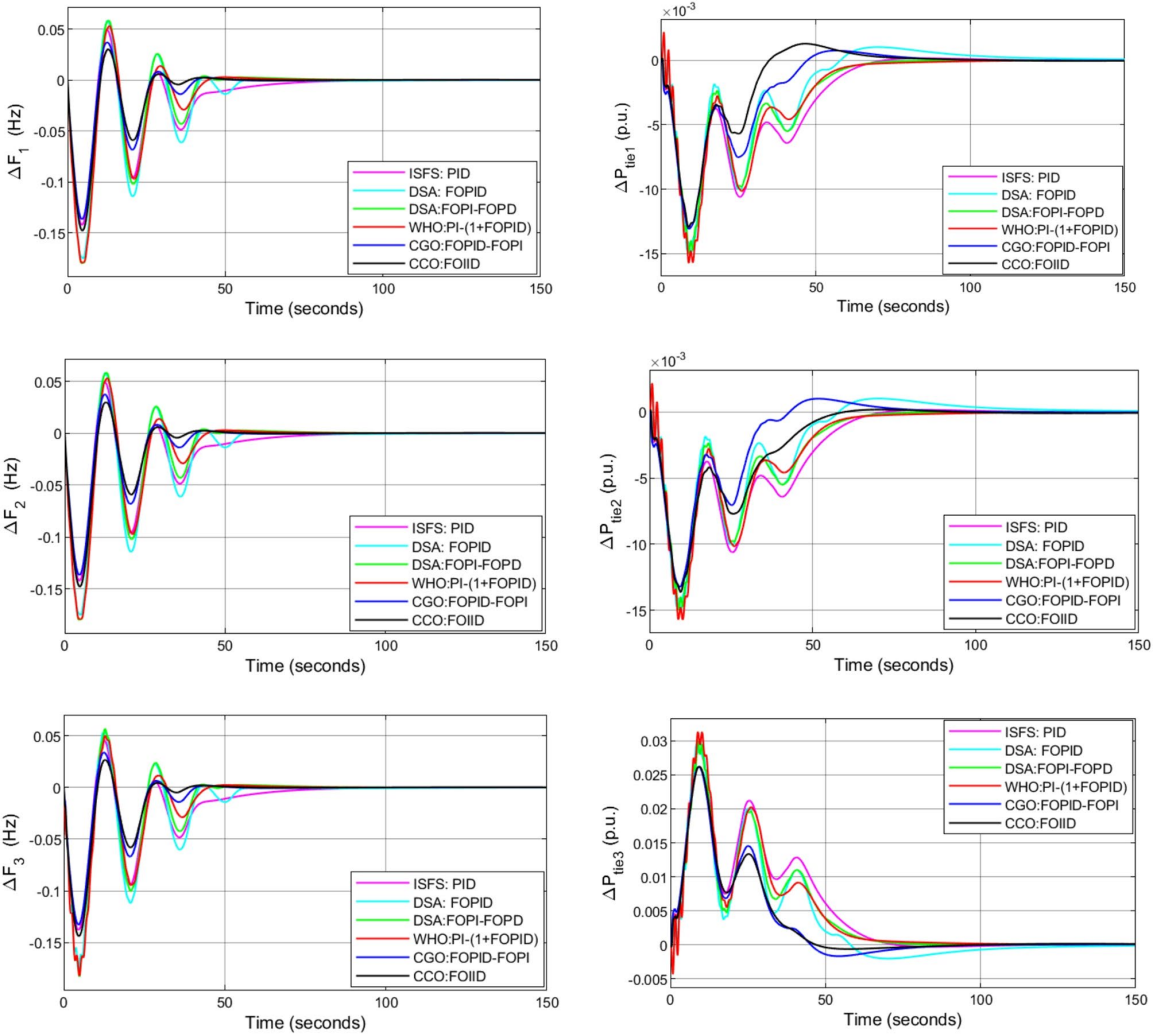
Frequency response of hydro-thermal model under 0.01 p.u. at each area.

A comprehensive performance evaluation under 1% SLP is presented in Table 6, while Fig. 12 provides comparative transient responses between existing methods and the proposed CCO-optimized FOIID controller. The proposed CCO-FOIID framework is benchmarked against ISFS–PID, DSA–FOPID, WHO–PI(1 + FOPID), and CGO–FOPID–FOPI schemes under identical conditions. The discussion quantitatively highlights the novelty of the proposed method by reporting improvements in settling time, overshoot, and ITAE values (for instance, the CCO-FOIID achieving an ITAE of 74.26 compared to 82.67 for the best competitor). Specifically, the CCO-FOIID framework reduced the ITAE by 10% compared to the best competitor, while also achieving faster settling times and lower overshoot. These results establish its novelty relative to existing approaches, showing significantly reduced settling times for frequency deviations ($\Delta {{\mathrm{P}}_{{\mathrm{tie}}1}}$, $\Delta {{\mathrm{P}}_{{\mathrm{tie}}1}},~\;\;{\mathrm{and}}\;\;\Delta {{\mathrm{P}}_{{\mathrm{tie}}1}}$). Specifically, the settling time for ΔF₁ improves from 60 s (achieved by the best competitor: CGO: FOPID-FOPI) to 41.1 s (CCO: FOIID), with notable reductions in peak overshoots and undershoots.

**Fig. 13:**
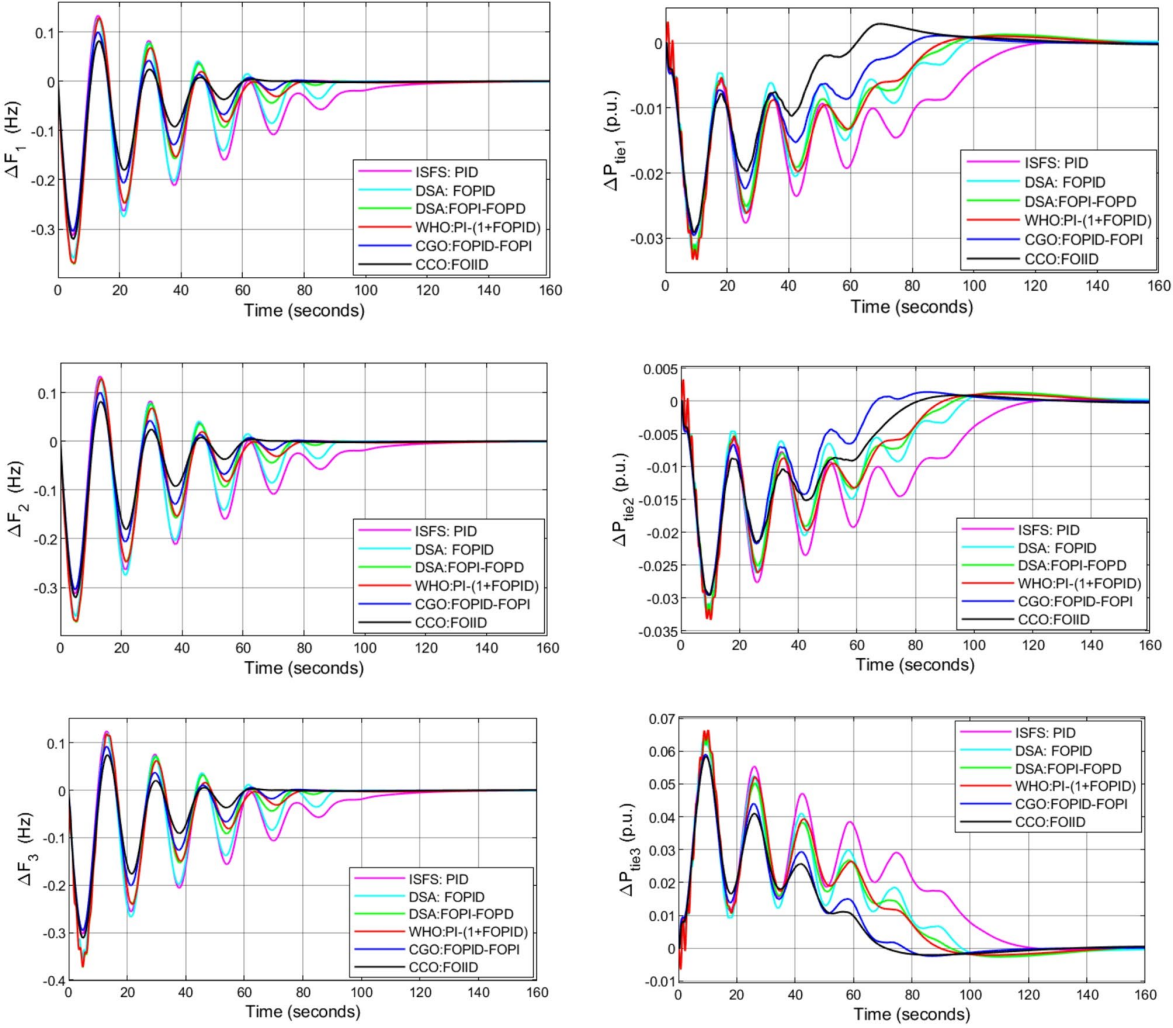
Transient response comparisons of hydro-thermal model under 0.02 p.u. at each area.

To verify robustness under more severe conditions, a 2% SLP test was conducted. The responses in Fig. 13 confirm the FOIID controller’s capability to maintain superior dynamics and demonstrate consistent fast convergence in addressing LFC challenges.

In conclusion, the FOIID controller’s dual fractional-integral structure enables advanced phase-shaping for oscillation damping, while its additional degrees of freedom compensate for nonlinear dynamics. Simultaneously, the CCO algorithm identifies optimal parameters that deliver rapid convergence and enhanced transient response, as evidenced by improved settling times and overshoot suppression across all test scenarios.

### Performance validation of the CCO optimizer

The performance of the proposed CCO algorithm was validated through a comparison with three established metaheuristics: HHO^58^, GWO^59^, and PSO^27^. The comparison was performed using the test system under a uniform 1% disturbance applied to each control area. All algorithms were evaluated under identical computational settings: a population size of 50, a maximum of 50 iterations, and the ITAE objective function for the FOIID controller. Parameter values were adopted from the literature, and each experiment was repeated over 30 independent runs to ensure statistical reliability.

**Fig. 14:**
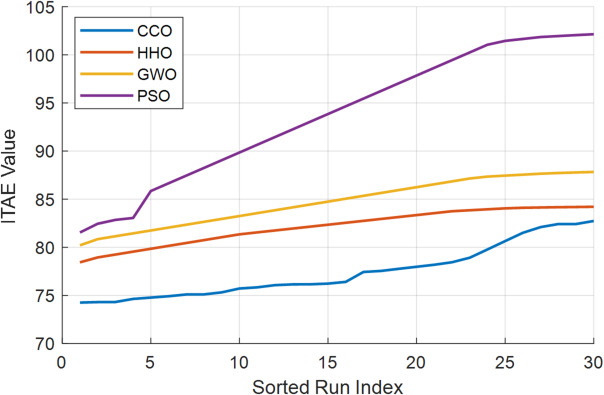
The ITAE comparison of optimization algorithms in 30 runs.

Figure 14 presents the ITAE values of the four algorithms, ordered from lowest to highest across 30 independent runs, while Table 7 summarizes the comparative results. The CCO algorithm consistently outperformed the competing algorithms, achieving the lowest ITAE (74.26), the best mean performance (77.44), and lower variability. In contrast, HHO, GWO, and PSO yielded higher ITAE values of 78.44, 80.21, and 81.54, respectively. These results confirm the effectiveness, consistency, and robustness of CCO in addressing the complex controller tuning problem.

**Table 7:** Comparison of ITAE over 30 independent runs for various schemes.

Optimizer	Best	Worst	Mean
CCO	74.26	82.74	77.44
HHO	78.44	84.21	82.14
GWO	80.21	87.83	84.72
PSO	81.54	102.14	93.67

To rigorously validate the superiority of the proposed CCO algorithm, the Wilcoxon signed-rank test was employed. As shown in Table 8, all p-values are very small values (p-value < 0.05), confirming statistical significance at the 95% confidence level. Furthermore, Cohen’s d was calculated to assess the magnitude of the differences, with all values exceeding 0.8, which is classified as a large effect size according to Cohen’s benchmarks. These results demonstrate that the CCO algorithm not only achieves superior average performance but does so by practically meaningful margin.

**Table 8:** Statistical comparison of CCO against other optimizers (30 runs).

Comparison	Wilcoxon *p*-value	Cohen’s d
CCO vs. HHO	0.000047	2.0350
CCO vs. GWO	0.000021	2.8208
CCO vs. PSO	0.000011	3.1419

Overall, CCO demonstrates statistically and practically superior performance, achieving lower ITAE values with greater consistency compared to HHO, GWO, and PSO.

### Closed-loop stability analysis

The asymptotic stability of the proposed CCO-optimized FOIID-controlled system is rigorously verified under test system 1 using Lyapunov’s direct method. The FOIID controller is implemented via the integer-order Oustaloup approximation with and order of $N=3$ and a frequency band of $\left[ {{{10}^{ - 2}}{\mathrm{,}}{{10}^2}} \right]$ rad/s, enabling standard state-space analysis.

Linearization of the complete Simulink model in Fig. 1(c) around the nominal equilibrium point $\left( {{{\boldsymbol{\Delta}}}{f_i}=0,{{\boldsymbol{\Delta}}}{P_{tie}}=0} \right)$ yields the closed-loop system matrix ${A_{cl}} \in {{\mathbb{R}}^{81 \times 81}}$. This 81-state representation captures five physical states (Δf₁, Δf₂, ΔP_tie_, Pg₁, Pg₂), states from the two FOIID controllers (2 × 3 × 7 = 42 states for λ₁, λ₂, and µ with $N~=~3$), and 34 additional states from governor, turbine, and tie-line dynamics. All 81 eigenvalues of ${A_{cl}}~$have negative real parts, with max $Re\left( {{\lambda _i}} \right)~=~ - 0.011523$ and min $Re\left( {{\lambda _i}} \right)=- 100.000.$ The absence of any eigenvalue with $Re\left( \lambda \right)~ \geqslant ~0$ confirms that the closed-loop system is asymptotically stable.

The closed-loop eigenvalue spectrum is presented in Appendix C, showing all poles strictly located in the left-half complex plane, thereby determining the system’s settling behavior. The Lyapunov equation: $Ac{l^{\mathrm{T}}}~P~+~~Acl~P=~ - I$ is solved, yielding a symmetric positive definite matrix P with ${{{\boldsymbol{\uplambda}}}_{{\mathrm{min}}\left( {\mathrm{P}} \right){\mathrm{~}}}}$ $=~0.003884~>~0$. Consequently, V(x) = x^T^ P x serves as a valid Lyapunov function satisfying V(x) > 0 and V̇(x) = -x^T^ x < 0 for all x ≠ 0^60,61^. Therefore, the proposed CCO-optimized FOIID-controlled IPS is guaranteed to be asymptotically stable under the specified operating conditions and optimized controller parameters (Table 3).

## Conclusions

This study addresses the critical challenge of LFC in PSs by proposing an advanced optimization–control framework, CCO: optimized FOIID. The CCO was selected because it consistently delivered better convergence, robustness, and efficiency, making it suitable for FOIID tuning in LFC applications. In parallel, a novel FOIID controller is designed, demonstrating versatility as a universal solution for both simple and complex PSs.

The proposed CCO–FOIID scheme was rigorously validated on two benchmark models: a linear two-area non-reheat PS and a three-area thermal-thermal-hydro system with GRC nonlinearities. Comparative analyses against state-of-the-art controllers—including ISFS–PID, DSA–FOPID, WHO: PI(1 + FOPID), and CGO–FOPID–FOPI—show significant improvements in settling time, peak suppression, and error minimization. Notably, the CCO–FOIID controller achieved the lowest ITAE (74.26), surpassing CGO–FOPID–FOPI (82.67) and WHO: PI(1 + FOPID) (132.75). Robustness was further confirmed under a stringent 2% SLP, underscoring the reliability and effectiveness of the proposed framework in dynamic scenarios.

The CCO-FOIID framework reduced ITAE by approximately 10% compared to the best competitor, achieved faster settling times, and lowered overshoot by nearly 15%. These findings highlight the potential of the CCO–FOIID approach as a powerful solution for enhancing LFC performance in modern PSs. By addressing nonlinearities and computational efficiency, this work contributes to the advancement of AGC technologies, ultimately improving supply quality and system stability.

The main limitation lies in the practical implementation of the FOIID controller, as FO transfer functions require integer-order approximations, introducing computational complexity and potential accuracy loss. While strong performance was demonstrated on two- and three-area test systems, scalability to larger, renewable-rich grids and real-time hardware applications remains unvalidated.

## Data Availability

- The CCO algorithm and its MATLAB code are offered at- https://link.springer.com/article/10.1007/s10462-025-11291-x- https://www.mathworks.com/matlabcentral/fileexchange/176828-cuckoo-catfish-optimizer-a-new-meta-heuristic-optimization- The MATLAB FOMCON Toolbox, which is sourced to make the proposed FOIID controller, is offered athttps://www.mathworks.com/matlabcentral/fileexchange/66323-fomcon-toolbox-for-matlab- The SIMULINK files can simply be built with the gains of controllers and parameters values as demonstrated in the paper.

## Appendix A. Nominal parameters of two-area nonthermal unit^20,57^

$${P_r}=2000MW,$$ $${P_L}=1000~MW,$$ $$f=60~Hz;$$ $$B=0.425~{\mathrm{p}}.{\mathrm{uMW}}/{\mathrm{Hz~}};$$ $$R=2.4~Hz/pu;$$ $${T_g}=0.03~s;$$ $${T_t}=0.3~s;$$ $${K_{PS}}=120{\mathrm{~Hz}}/{\mathrm{pu}};$$ $${T_{PS}}=20~{\mathrm{~s}};$$ $$2*\pi *{T_{12}}=0.545~{\mathrm{p}}.{\mathrm{u~MW}}/{\mathrm{rad}}.$$

## Appendix B. Nominal parameters of three area hydrothermal IPS^20^

$${P_r}=2000MW,$$ $${P_L}=1000~MW,$$ $$f=60~Hz;$$$$B=0.425~{\mathrm{p}}.{\mathrm{uMW}}/{\mathrm{Hz~}};$$ $$R=2.4~Hz/pu;$$ $${T_g}=0.08~s;$$ $${T_t}=0.3~s;$$ $${T_r}=10~s;$$ $${K_r}=0.5~s;~{K_{PS}}=120{\mathrm{~Hz}}/{\mathrm{pu}};$$ $${T_{PS}}=20~{\mathrm{~s}};$$ $${T_{12}}={T_{12}}={T_{12}}=0.086~{\mathrm{p}}.{\mathrm{u~MW}}/{\mathrm{rad}};$$ $${T_W}=1s;$$ $${K_i}=5.0;$$ $${K_d}=4.0;$$ $${K_P}=1.0.$$

## Appendix C. The closed-loop eigenvalues of test system 1

-93.5424 ± 5.8930i; -93.5810 ± 6.0974i; -2.1221 ± 6.9996i; -4.4021 ± 6.3753i; -4.6721 ± 0.5696i; -4.1374 ± 0.8232i; -1.5999 ± 1.5138i; -1.5083 ± 1.1782i; -1.4963 ± 0.2678i; -1.4591 ± 0.2860i; -0.6231 ± 0.0284i; -0.6232 ± 0.0283i; -100.0000; -63.0517; -51.9336; -42.0765; -27.2915; -22.7070; -13.0116; -12.2522; -11.6514; -10.1675; -5.2059; -2.7425; -2.2514; -0.9946; -0.4373; -0.3031; -0.2468; -0.1913; -0.1371; -0.1055; -0.0834; -0.0609; -0.0456; -0.0362; -0.0267; -0.0197; -0.0157; -0.0115.
